# Applicability to foraging simulation of a reinforcement schedule controlling the response energy of pigeons

**DOI:** 10.3758/s13420-013-0117-7

**Published:** 2013-08-15

**Authors:** Masanori Kono

**Affiliations:** Department of Psychology, Meisei University, 2-1-1 Hodokubo, Hino-city, Tokyo 191-8506 Japan

**Keywords:** Optimal foraging theory, Energy expenditure, Interresponse energy, Pigeon

## Abstract

In optimal foraging theory (OFT), energy expenditure is an important variable for predicting foraging behavior. However, early studies, including operant simulations of foraging, did not measure energy expenditure. In the present study, an adjusting energy (AE) schedule was developed to control energy expenditure. Interresponse energy (IRE), a measure of the energy expenditure during a response, was calculated by dividing the square of the elapsed time between two consecutive responses by the square of the straight-line distance between the locations of the same two responses. An adjusting procedure was employed to estimate the indifference point between the requirements of the AE schedule and a fixed ratio (FR) schedule, which has been used in many operant simulations. In the adjusting procedure, pigeons adjusted the requirement of the AE schedule to that of the FR schedule. The results showed a systematic relationship between the requirements of the AE and FR schedules. Moreover, the total IRE per reinforcement systematically increased with the AE requirement. Thus, the present study demonstrates the utility of the AE schedule as a procedure for testing the validity of OFT.

The foraging behavior of animals has received attention for many years (Lea, 1981; Shettleworth, 1988, 1989). Many studies have attempted to investigate foraging behavior by quantitative analysis (Stephens & Krebs, 1986). The optimal foraging theory (OFT), which is formulated on the basis of the concept of optimization, has been a valuable tool for the quantitative analysis of foraging behavior (Charnov, 1976a, 1976b; Davies, 1977; Emlen, 1966; Gendon, 1987; Kacelnik, 1984; MacArthur & Pianka, 1966). The basic prediction of the OFT is that organisms will forage in such a way that the rate of energy intake is maximized.

Energy intake is expressed by the relationship between energy gain and energy expenditure. Energy gain is the amount of energy obtained from prey, whereas energy expenditure is the amount of energy expended by foraging behavior, such as moving to a patch and searching for or handling prey. Thus, OFT predicts an animal’s foraging behavior on the basis of the energy gain and energy expenditure spent in foraging. Therefore, in order to investigate the validity of OFT, energy gain and expenditure must be determined. However, relatively few studies have addressed this question, and those that have have been done by researchers in both biology (e.g., Elliot, Davoren, & Gaston, 2008; Liu, Bernstein, & Thiel, 2009; Tieleman, Dijkstra, Klasing, Visser, & Williams, 2008) and psychology (Aparicio & Baum, 1997; Cassini, Kacelnik, & Segura, 1990; Kacelnik & Todd, 1992; Kono & Omino, 2008; Lea, 1979; Mitchell & Brener, 1991; Redhead & Tyler, 1988).

Simulations employing operant techniques have been used most frequently in research on foraging. The operant simulations have gained recognition in both biology and psychology, because they enable carefully controlled procedures (Aparicio & Baum, 1997; Dallery & Baum, 1991; Lea, 1981; Shettleworth, 1988; Stephens & Krebs, 1986). For example, many operant simulations have used ratio schedules, in which the last of a specified number of responses is reinforced, to model various aspects of the foraging behavior (e.g., Dallery & Baum, 1991; Dow & Lea, 1987; Kono & Omino, 2008). Operant simulations using ratio schedules have defined the quantity of food as the energy intake and the number of responses emitted under the ratio schedules as the energy expenditure. Thus, they have made it possible to quantitatively control the amount of energy, to some extent.

Additionally, the utility of the barrier-choice procedures has been suggested by previous studies (Aparicio, 1999, 2001; Aparicio & Baum, 1997). In barrier-choice procedures, the subjects must climb over a barrier to switch from one alternative to another. For example, Aparicio (2001) manipulated the effort required in locomotion to travel from one lever to another by changing the height of the barrier. Thus, the barrier-choice procedures straightforwardly controlled energy expenditure, because they involved the locomotion of the entire body, which is similar to the behavior in the natural foraging situation.

As was stated above, many studies have suggested that operant simulations are a suitable means of testing the OFT (Aparicio & Baum, 1997; Dallery & Baum, 1991; Lea, 1981; Shettleworth, 1988; Stephens & Krebs, 1986). However, previous studies of operant simulations have had limitations for quantitative evaluation of the validity of OFT. Most of the operant simulations, either the ratio schedules or the barrier-choice procedures, have measured the number or the time of responses as a proxy for energy expenditure (Aparicio, 1999, 2001; Aparicio & Baum, 1997; Dow & Lea, 1987; Kono & Omino, 2008). Although the validity of these variables as a surrogate for energy expenditure has been suggested (Dallery & Baum, 1991), the number or time of responses cannot be directly converted to energy expenditure, because the quantitative relationship between the number or time of responses and energy expenditure has not been determined. Therefore, in order to investigate the validity of OFT more rigorously, it was necessary to develop a procedure in which the controlling variable was energy expenditure for operant behavior.

The joule is defined as the energy expended in applying a force of one newton through a distance of one meter (McGlashan, 1971), as follows:

1 $$ 1\ \mathrm{J}=1\ \mathrm{N}\cdot \mathrm{m}. $$

The newton is defined as the amount of net force required to accelerate a mass of one kilogram at a rate of one meter per second squared (McGlashan, 1971), as follows:

2 $$ 1\ \mathrm{N}=1\ \mathrm{kg}\cdot \mathrm{m}/{\mathrm{s}}^2. $$

By substituting Eq. 2 into Eq. 1, the following equation is obtained:

3 $$ 1\ \mathrm{J}=1\ \mathrm{kg}\cdot {\mathrm{m}}^2/{\mathrm{s}}^2. $$

Therefore, the joule can also be defined by mass, distance, and time. These parameters can be regarded as the body mass of an animal (in kilograms), the movement distance of behavior (in meters), and the elapsed time of behavior (in seconds). Thus, these are measurable quantities in the operant simulation. In the present study, the energy expenditure for operant behavior is defined as follows:

4 $$ E=M\cdot {D}^2/{T}^2, $$

where *E* is the energy expenditure (in joules), *M* is an animal’s mass (in kilograms), *D* is the distance moved (in meters), and *T* is the elapsed time (in seconds). It is necessary to measure *M*, regardless of whether *E* is calculated for a part of or for the entire body. However, in the single-subject design of operant simulation, the body mass of subjects is typically maintained at an approximately constant level throughout each experimental condition. Here, we make the assumption that *M* is constant for the same subject throughout an experiment. Thus, *E* can be calculated by determining *D* and *T*.

Developing a reinforcement schedule that controls energy expenditure would make it possible to simulate the foraging behavior in a natural environment more strictly and to test quantitatively the validity of OFT. Therefore, as a procedure for testing OFT, the present study was designed to explore the utility of the energy schedule in which reinforcement occurred only when the energy expenditure of responses was greater than some specified requirement. Moreover, the indifference point between the requirements of the fixed-ratio (FR) and energy schedules was estimated by using an adjusting procedure (e.g., Mazur, 1987), in order to investigate the functional relationship between energy expenditure and the FR requirement. The present study used the FR schedule as a representative of ratio schedules. This is because many studies have used the FR schedule to simulate the various components of foraging behavior, such as searching for prey (Dow & Lea, 1987), choosing prey (Abarca & Fantino, 1982), and traveling between patches (Kono & Omino, 2008).

## Method

### Subjects

Three homing pigeons (MP102, MP701, and MP702) served as subjects. MP102 had previously been trained on a variety of reinforcement schedules, whereas MP701 and MP702 were experimentally naive. The naive pigeons were trained to peck a response key by using successive approximations. During the experimental sessions, all subjects were maintained at 80 % of their free-feeding weights. They received mixed grain outside the experimental chamber in order to maintain their weight, were housed individually in cages, and had free access to water.

### Apparatus

The experiment was conducted in an operant chamber measuring 38.0 cm high, 51.0 cm wide, and 51.0 cm long. The front wall contained a circular response area (22-cm diameter) that gave access to a liquid crystal display monitor with a touch panel (Gunze, AV7629FT03). The circular response area was centered horizontally on the wall, 21.0 cm above the floor. The entirety of the circular response area could be illuminated with either red or blue light. Two response keys (5-cm diameter) were also displayed in the circular response area. The left and right keys were blue and red, respectively. The centers of the keys were separated by a distance of 10.0 cm and were 20.0 cm above the floor. A force of approximately 0.01 N was required to operate the touch panel. A standard hopper, accessible through a rectangular opening (4.5 cm high and 5.5 cm wide) in the lower center of the rear wall of the chamber, provided 4-s accesses to hemp seeds as reinforcers. When the reinforcers were delivered, the response area was dark and the white light above the hopper was lit. The procedural events and data recording were controlled using a personal computer (Dell, Latitude D610). Continuous white noise was provided by a white noise generator located outside the chamber.

### Procedure

**Fig. 1:**
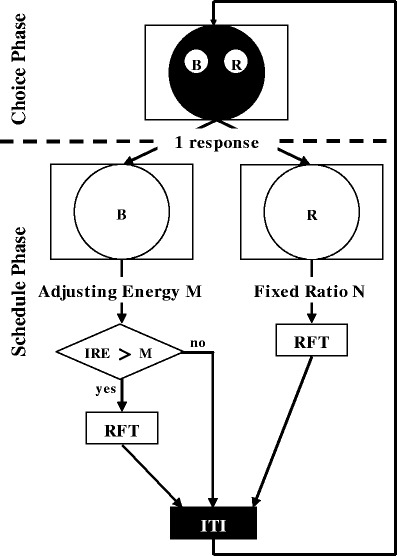
Schematic diagram of the procedure for free-choice trials. “B” and “R” at the response keys or response areas refer to blue and red, respectively. The procedure for the forced-choice trials was the same as that for the free-choice trials, except that only one key was presented during the choice phase

Experimental sessions were conducted daily. The sessions ended after 60 trials or when 90 min had elapsed, whichever came first. The adjusting procedure consisted of the choice phase and the schedule phase (Fig. 1). The FR and adjusting energy (AE) schedules were arranged at the schedule phase. In these schedules, the subjects could peck anywhere on the circular response area, which was illuminated with either red or blue light. In the FR schedule, the entirety of the circular response area was illuminated with red light. After the subjects had pecked anywhere on the circular response area several times, as required for the FR schedule, the response area was turned off and reinforcement followed. In the AE schedule, the entirety of the circular response area was illuminated with blue light. The subjects were required to peck anywhere on the response area twice. The first response turned the color of the circular response area from blue to white, and the second response turned off the response area light. If the interresponse energy [IRE (in joules)] calculated for these two responses was greater than the AE requirement, reinforcement was followed by the second response. If the IRE was less than the AE requirement, a 3-s intertrial interval (ITI) was followed by the second response. The IRE was defined by the distance and time of responses, as was mentioned in the introduction. The distances of the responses were measured as the interresponse distance [IRD (in meters)], which was the straight-line distance between the locations of the first and second responses. Response times were measured as the interresponse time [IRT (in seconds)], which was the elapsed time between the first and second responses. The IRE was calculated as follows:

5 $$ \mathrm{IRE}={\mathrm{IRD}}^2/{\mathrm{IRT}}^2. $$

Equation 5 indicates that the number of combinations of IRT and IRD that met a given AE requirement was infinite. If, for example, the AE requirement was 0.25 J and the IRT was 0.1 s, then the IRD must be greater than 0.05 m to meet the requirement. Similarly, if the IRT was 0.2 s, then the IRD must be greater than 0.1 m to meet the requirement, and so on.

The 60 trials were divided into 15 blocks of four trials each. The first two trials of each block were forced-choice trials, in which only one key was displayed in the circular response area during the choice phase. The last two trials of each block were free-choice trials, in which both keys were displayed. Figure 1 shows the procedure for the free-choice trials. Here, two response keys were displayed in the circular response area at the beginning of each trial. A single peck on either response key extinguished both keys, and the entirety of the response area was illuminated by the color of the selected key. A peck on the right key (red) led to the FR schedule, whereas a peck on the left key (blue) led to the AE schedule. After completing these schedules, reinforcement occurred, and a 3-s ITI followed. During the ITI, the circular response area was turned off. After the ITI, the next trial began.

The procedures for the forced-choice and free-choice trials were the same, except that only one key was displayed in the circular response area during the choice phase, and the correction method was applied to the AE schedule during the schedule phase. If the IRE was less than the AE requirement, then the AE schedule, but not the choice phase of the next trial, began as a correction trial, again after the ITI. The maximum number of correction trials was set as five in order to avoid extinguishing the subject’s response. If the IRE was less than the AE requirement in the fifth correction trial, the ITI began, and then the choice phase of the next trial followed. Of every two forced-choice trials, one involved the left key and the other the right, determined pseudorandomly.

The AE requirement depended on the subject’s previous choice. If the subject chose the left key on both free-choice trials, the AE requirement was increased by 0.001 J for the next block of trials. If the subject chose the right key on both free-choice trials, the AE requirement was decreased by 0.001 J for the next block of trials. If the subject chose each key once, the AE requirement was unchanged. These rules for adjusting the AE requirement were applied from the last block of one session to the first block of the next session. After the first session, the starting requirement of the AE schedule in the subsequent session was determined on the basis of the choices in the final block of the preceding session.

The FR requirement was 15, 30, or 60; a given requirement was constant throughout a condition. The order of the conditions was an ascending and then descending series of the FR requirements: 15, 30, 60, 30, and 15 (Table 1). All conditions lasted for a minimum of 18 sessions. To assess stability, the mean AE requirement was calculated for each session. After the minimum number of sessions, a condition was terminated when several stability criteria were met, as follows: (1) Neither the highest nor the lowest mean AE requirement of a condition could occur in the last six sessions of a condition; (2) no systematic upward or downward trends in the mean AE requirement appeared over the last six sessions; and (3) the mean AE requirement calculated over the last six sessions did not differ from that of the preceding six sessions by more than 7.5 %.

**Table 1:** Numbers of sessions for each condition

Condition	MP102	MP701	MP702
FR 15	28	41	61
FR 30	30	18	42
FR 60	27	45	21
FR 30	36	39	24
FR 15	20	44	27

## Results

**Fig. 2:**
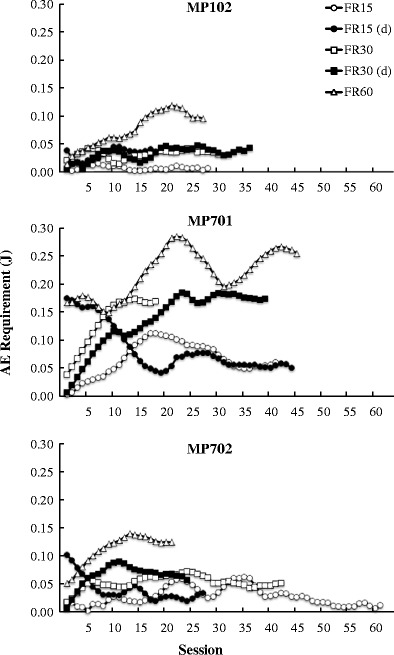
Adjusted energy (AE) requirements, plotted session by session for each subject. Open symbols indicate the results from ascending series, and filled symbols indicate those from descending series

Figure 2 shows the variation in the AE requirements across all sessions for each condition. The open symbols indicate the results from the ascending series, and the filled symbols indicate those from the descending series. The results for subjects MP102 and MP702 showed that the AE requirements became stable after approximately 15 sessions. On the other hand, the results for MP701, particularly for FR 60, showed relatively large fluctuations after 15 sessions. However, these fluctuations decreased as the sessions continued, and the AE requirements eventually became stable. For all subjects, the numbers of sessions required until the AE requirement became stable did not systematically change with the FR requirement.

**Fig. 3:**
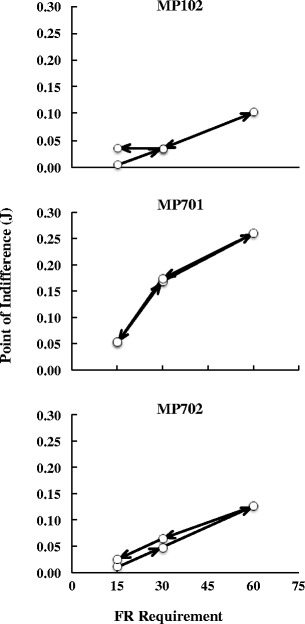
Indifference points of the AE requirements as a function of the fixed-ratio requirements. Arrows indicate the order of conditions

Figure 3 shows the indifference points, plotted as a function of the FR requirement for each subject. In this experiment, the subjects were required to adjust the AE requirement depending on the FR requirement. Therefore, the indifference point was defined as the mean AE requirement calculated over the last six sessions for each FR requirement. Open symbols indicate the indifference points, and arrows indicate the order of conditions. The indifference points systematically increased as the FR requirement increased for all subjects. A Friedman test showed a significant effect of the FR requirement, *χ* ^2^ = 8.4, *df* = 2, *p* < .05. In particular, MP102 and MP702 showed a linear increase and similar requirements at each FR requirement. Similarly, MP701 showed a systematic, but nonlinear, increase. The indifference points for MP701 were higher than those for the other subjects at each FR requirement. The indifference points for the ascending series were nearly equal to those for the descending series, except for the results from FR 15 for MP102.

**Fig. 4:**
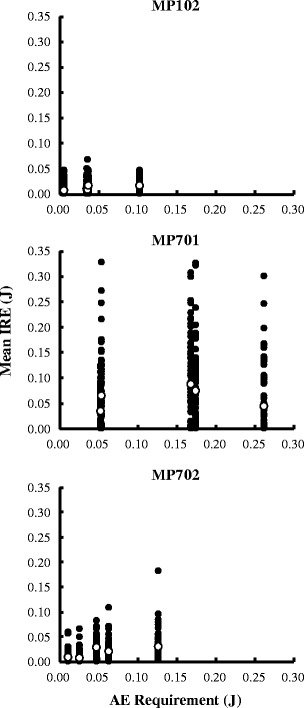
Interresponse energy (IRE) as a function of the AE requirement. Filled symbols indicate one IRE, and open symbols indicate the mean IRE

**Fig. 5:**
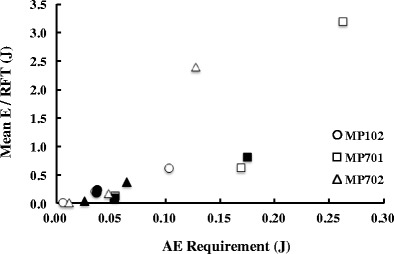
Mean energy per reinforcement (*E*/RFT) as a function of the AE schedule. Open symbols indicate the data from ascending series, and filled symbols indicate those from descending series

Figures 4 and 5 represent the subjects’ performances in the AE schedules. The data displayed in these figures are based on the results of the free-choice trials of the last six sessions for each condition. The *x*-axes of Figs. 4 and 5 indicate the AE requirements. In this experiment, the AE requirement could change between trials because of the adjusting procedure; however, the AE requirements on the *x*-axis were defined as the mean AE requirements calculated over the last six sessions, which were thus the indifference points (Fig. 3). For example, the AE requirements for MP702 were 0.011, 0.025, 0.047, 0.064, and 0.127 J. Therefore, these are the values on the *x*-axis for MP702 in the following figures.

Figure 4 shows the distribution and the mean IRE as a function of the AE requirement for each subject. Each filled symbol indicates a single IRE, and open symbols indicate the mean. The distributions of the IRE differed between subjects. The data for MP102 showed that the distribution of the IRE remained unchanged with increasing AE. Furthermore, the distribution of the IRE for MP701 did not systematically change with the AE requirement; however, the range of the distribution for MP701 was large, as compared with those of the other subjects. MP702 showed a range of the distribution that increased slightly as the AE increased. Across subjects, the mean IRE did not change systematically with AE requirements.

In the AE schedule, the number of trials for each reinforcement could vary between conditions or subjects, because responses were not reinforced on trials for which the IRE was less than the AE requirement. Thus, even if the mean IREs were equal for the AE requirements (Fig. 4), the total amounts of the IRE for each reinforcement would be different if the numbers of trials on which reinforcement occurred were different between the AE requirements. Thus, the total energy per reinforcement (*E*/RFT) for the AE schedule was calculated by summing the IREs of all responses that occurred for a given reinforcement. Because no reinforcement occurred at AE 0.103 J for MP102, the *E*/RFT in this case was calculated as the total IRE. Figure 5 shows *E*/RFT as a function of the AE requirements for individual pigeons. For all pigeons, the *E*/RFT systematically increased with the AE requirements. A significant correlation emerged between the *E*/RFT and the AE requirement (*r* = .83, *p* < .05).

## Discussion

The present study was designed to investigate the indifference point between the requirements of the FR schedule and the schedule of reinforcement in which energy expenditure was the controlling variable. Figure 3 shows that for the pigeons, the indifference points systematically increased as the FR requirement increased, although there were individual differences. Additionally, the descending series of the experiment confirmed the results of the ascending series. These data suggest that there is a systematic relationship between choices of the AE and FR requirements. This indicates the possibility that the functional relationship between energy expenditure and FR requirement could be estimated. However, in the present study, a quantitative analysis of the relationship could not be conducted, because of the limited data obtained from the experiment involving only three conditions. In particular, a limited range of FR requirements were used, in accordance with the range used in published studies that had employed the FR schedule (Kacelnik & Todd, 1992; Kono & Omino, 2008). Therefore, future studies should use a broader range of FR requirements to investigate the quantitative relationship between the AE and FR requirements.

The present study developed an AE schedule to control energy expenditure. To investigate the effect of the AE on the energy expenditure for each response, the mean IRE should be analyzed. As is shown in Fig. 4, the mean IRE did not increase as the AE requirement increased, and only two of 15 data points exceeded the AE requirements. Therefore, the effects of the AE requirement on the IRE were not so strong that we could consider that the AE schedule controlled the energy expenditure. This result appears to be affected by variation in the requirements of the AE schedule. In the present study, the AE requirement changed between trials or between sessions (Fig. 2) because of the adjusting procedure. Studies by others have suggested that variation in schedule requirements affects performance under the schedule of reinforcement (e.g., Ferster & Skinner, 1957; Kramer & Rilling, 1970; Lee, Sturmey, & Fields, 2007). For example, under a mixed differential-reinforcement-of-low-rate schedule in which two IRTs were reinforced, the shorter IRT was preferred over the longer (Kramer & Rilling, 1970). Similarly, the present results may have been affected by the strong effect of a shorter AE requirement. As is discussed below, however, the weak effect of the AE schedule on the IRE of each response does not present a significant issue with respect to the utility of the AE as a procedure for foraging simulation.

Figure 5 shows that the *E*/RFT increased with the AE requirement for all subjects. This resulted from the fact that the number of error trials increased with the AE requirement. In the AE schedule, when the subjects failed to meet the AE requirement, reinforcement did not occur. *E*/RFT was calculated on the basis of the IREs of all responses, including the IREs spent on responses emitted on trials without a reinforcer. Therefore, even if the IRE of each response does not change as the AE requirement changes, the increase in the number of trials on which reinforcement occurs causes *E*/RFT to increase as a function of the AE requirement. Considering that the mean IRE did not change appreciably with the AE requirement, the systematic increase in the *E*/RFT reflected an increase in the number of trials without reinforcement.

Although the effects of the AE schedule on the IRE were not strong, the increase in the *E*/RFT with AE requirement implies the applicability of the AE schedule with respect to the foraging simulation procedure. OFT predicts an animal’s foraging behavior on the basis of the total amount of energy expenditure required to complete the behavioral unit, which is the entirety of the responses until the objective is accomplished, such as searching for prey, procurement of prey, or arriving at a new patch (e.g., Charnov, 1976a, 1976b; Emlen, 1966). For example, when OFT is used to predict the effect of the energy expenditure to move to a new patch, the entire sequence of walking to the patch may be included in the analysis, but each step of the walking as a part of the whole sequence may not (Charnov, 1976b). Therefore, a procedure that produces an increase in the total energy expenditure is required in order to investigate the validity of OFT. Thus, the AE schedule developed here is a suitable procedure for testing OFT.

In the present study, an AE schedule was developed as a procedure to control the energy expenditure of behavior. Considering that OFT is formulated on the basis of energy expenditure (e.g., Charnov, 1976a, 1976b; Emlen, 1966) and that the total energy per one reinforcement increased as a function of the AE requirement (Fig. 5), the present study supports the utility of the AE schedule for testing the validity of OFT. However, the effect of the AE requirement on each response was not as strong as those of distance or effort that had been obtained in previous studies (e.g., Aparicio, 1999, 2001; Aparicio & Baum, 1997; Reilly, Posadas-Sanchez, Kettle, & Killeen, 2012). Therefore, further study will be necessary to modify the AE schedule, such that it will be able to more strongly control the IRE. By doing so, it will be possible to quantitatively investigate the validity of OFT.
